# Volatile sulfide compounds and oral microorganisms on the inner surface of masks in individuals with halitosis during COVID-19 pandemic

**DOI:** 10.1038/s41598-023-29080-3

**Published:** 2023-02-13

**Authors:** Yeon-Hee Lee, Hyeongrok Kim, Dae Wook Heo, In-Suk Ahn, Q-Schick Auh

**Affiliations:** 1 Department of Orofacial Pain and Oral Medicine, Kyung Hee University School of Dentistry, Kyung Hee University Medical Center, #613 Hoegi-Dong, Dongdaemun-Gu, Seoul, 02447 South Korea; 2 Life Science Institute, Denomics Inc. 518, 5 Digital-Ro 26-Gil, Guro-Gu, Seoul, 08389 South Korea

**Keywords:** Microbiology, Biomarkers, Health care, Medical research, Risk factors

## Abstract

Mask-wearing is still recommended owing to the continuing impact of the COVID-19 pandemic. Within the closed chamber created by the mask, people are increasingly self-aware of their oral malodor. In this prospective and cross-sectional study, we aimed to measure volatile sulfide compound (VSC) levels in patients with halitosis and investigate the oral microbiome profile on the inner surface of their KF94 masks. We also investigated which oral microbiota increases VSC levels and whether the oral microbiomes of oral saliva and mask are correlated. A total of 50 subjects (41 women, average age 38.12 ± 12.58 years old) were included in the study, 25 healthy subjects and 25 patients with halitosis who wore masks for more than 3 h. The dominant bacterial species, bacterial profile, and Shannon diversity index of whole unstimulated saliva and the inner surface of the mask were investigated. The bacterial 16S ribosomal RNA genes of the major oral bacterial species were analyzed using real-time PCR. Gas chromatography was used to measure hydrogen sulfide (H_2_S) and methyl mercaptan (CH_3_SH), which are representative VSCs. The total bacterial DNA copy number was significantly higher in the saliva sample than in the mask sample (*p* < 0.001), and the average value was 276 times greater. Shannon diversity index was also significantly higher in saliva than in the inner surface of the mask (2.62 ± 0.81 vs. 1.15 ± 1.52, *p* < 0.001). The most common Gram-negative and Gram-positive species in the masks were *Porphyromonas gingivalis* (*Pg*) and *Lactobacillus casei* (*Lc*), respectively. The bacterial species with significant positive correlations between saliva and mask samples were *Prevotella intermedia* (*Pi*) (r = 0.324, *p* = 0.022), *Eikenella corrodens* (r = 0.309, *p* = 0.029), *Lc* (r = 0.293, *p* = 0.039), and *Parvimonas micra* (*Pm*) (r = 0.366, *p* = 0.009). The mean value of CH_3_SH was significantly higher in the halitosis group than in the non-halitosis group (17.84 ± 29.00 vs. 3.84 ± 10.57 ppb, *p* = 0.031). In the halitosis group, the DNA copy numbers and VSC levels showed highly positive correlation coefficients in the order *Pg*, *Treponema denticola (Td)*, *Tannerella forsythia (Tf)*, *Pi*, and *Prevotella nigrescens* (*Pn*) (all *p* < 0.05). Regarding bacterial profiles of the mask, *Td* was strongly correlated with CH_3_SH (r = 0.414, *p* = 0.040) and total VSCs (r = 0.374, *p* = 0.033) only in halitosis group. Mask-wearing time was strongly correlated with total VSCs, H_2_S, and CH_3_SH (all r > 0.8, *p* < 0.001). Oral bacteria, whose association with halitosis has been identified, increased VSC levels in mask-wearing subjects during the COVID-19 pandemic, particularly the number of Gram-negative anaerobes such as *Pg* and *Td*. Mask-wearing time was a major factor in increasing VSC levels. The study results suggest that people with halitosis could control these Gram-negative bacteria by improving oral hygiene and regularly changing masks.

## Introduction

Halitosis, also called oral malodor, is a common condition. The term “halitosis” is derived from the Latin *halitus* (breathed air) and Greek *osis* (pathological alteration)^1^. Halitosis is prevalent in up to 60% of the human population worldwide^2^. During the Coronavirus disease 2019 (COVID-19) pandemic, the prevalence of halitosis was 32.0% when wearing masks^3^. Halitosis is a common complaint among both sexes and all age groups^4^. In 90% of patients, halitosis originates from the oral cavity, primarily due to poor oral hygiene, tongue coating, dental biofilm, dental caries, oral mucosal diseases, and periodontal diseases^5^. However, 9% of the causes of halitosis are non-oral, such as inflammatory reactions around the oral cavity, rhinitis, and maxillary sinusitis, and respiratory, gastrointestinal, and urinary problems^6,7^. In 1% of patients, halitosis is caused by diet or drug metabolism^7^. Halitosis is a condition that has plagued mankind since ancient times, and mask-wearing has become routine due to the COVID-19 pandemic.

COVID-19, which was reported in December 2019, was declared an official pandemic by the World Health Organization (WHO) on January 30, 2020. Owing to the exponential spread of COVID-19 worldwide and its devastating impact, the WHO had recommended regulations to stop the spread of COVID-19^8^. Mask-wearing was strongly recommended during the COVID-19 lockdown period. Guidelines for the general public from the Center for Disease Control, WHO, included wearing face masks to minimize the risk of transmission of the SARS-CoV-2 virus^9^. However, a tight mask can induce a hypercapnic hypoxic environment with inadequate oxygen and carbon dioxide (CO_2_) exchange^10^. Thus, inside the mask, CO_2_ may accumulate and create a humid anaerobic environment. Moreover, the mask forms a closed circuit for inhalation and exhalation. Rebreathing exhaled gas increases the acidity levels inside the mask. Inhaling high levels of CO_2_ causes headaches and may be life threatening^11^. Furthermore, the chamber created by the mask with the mouth, nose, and skin can cause high humidity and temperature rise. A moist and anaerobic environment favors the growth and reproduction of microorganisms, especially Gram-negative anaerobes^12^.

Halitosis is primarily caused by volatile compounds, which are produced by the putrefactive action of both aerobic and anaerobic oral microorganisms on endogenous or exogenous proteins and peptides^2^. In particular, Gram-negative anaerobes tend to produce foul-smelling sulfur-containing gases called volatile sulfur compounds (VSCs)^13^. Representative oral bacteria related to halitosis are *Prevotella spp., Porphyromonas spp., Tannerella forsythia, Fusobacterium spp.*, *Actinomyces spp.*, *Bacteroides spp*., and *Eubacterium spp*^2,14,15^*.* The essential VSCs are hydrogen sulfide (H_2_S), methyl mercaptan (CH_3_SH), and dimethyl sulfide^16^. Hydrogen sulfide, methyl mercaptan, and to a lesser extent, dimethyl sulfide, represent 90% of the VSCs in halitosis^17^. Gas chromatography is the preferred technique to detect and measure VSCs^18^. Halitosis is sometimes classified as genuine halitosis, in which VSCs are detected above a significant level, and subjective pseudo-halitosis, in which VSCs are not detected^19^. In this study, the oral microbial profiles of saliva and inner surfaces of the mask of individuals with self-perceived halitosis were investigated and compared with those of healthy controls without halitosis. In addition, the VSCs levels of all the participants were measured directly using gas chromatography.

The hypothesis of this study is that (1) prolonged wearing of masks in the context of the COVID-19 pandemic will adversely affect the level of bad breath in individuals with self-perceived halitosis; (2) microorganisms may also be present inside masks, including saliva-derived oral microorganisms, which grow after attaching to the inner surface of the mask; (3) oral microorganisms from saliva may be a major factor in halitosis, and some microorganisms in mask samples may also affect halitosis. To our knowledge, the present study is the first to investigate oral microorganisms in the saliva and mask inner surfaces of individuals with self-perceived halitosis. This study aimed to comprehensively investigate the clinical characteristics and VSC levels using gas chromatography, and to clarify the factors causing the increase in VSC levels. Although halitosis is not usually life-threatening and fatal, it is common in COVID-19 patients wearing masks and can disrupt interpersonal social communication and relationships, cause psychological disturbances, and lower the quality of life. Therefore, elucidating the relationship between mask-wearing and halitosis, and investigating whether oral microorganisms cause halitosis, are important in the context of the COVID-19 pandemic.

## Results

### Demographics and salivary characteristics

The average mask wearing time of 50 participants (41 women, mean age; 38.12 ± 12.58 years) was 5.88 ± 2.93 h. The age or sex composition did not differ significantly between the non-halitosis and halitosis groups. Mask-wearing time, smoking status, and candidiasis status did not significantly differ between the halitosis and non-halitosis groups. As the mean values of UFR in non-halitosis (1.05 ± 0.25 mL/min) and halitosis (1.06 ± 0.40 mL/min) groups were not significantly different (*p* > 0.05), the presence of xerostomia was significantly higher in the halitosis group than in the non-halitosis group (44.0 vs 12.0%, *p* = 0.025) (Table 1). Salivary pH (7.06 ± 0.42 vs. 7.19 ± 0.40) and buffer capacity (10.24 ± 0.83 vs. 9.88 ± 0.88) were also not significantly different between non-halitosis and halitosis groups (all *p* > 0.05).

**Table 1:** Demographics, clinical characteristics, and volatile sulfide compounds level.

	Total (n = 50)	Non-Halitosis (n = 25)	Halitosis (n = 25)	*p* value
	Mean ± SD or n (%)	Mean ± SD or n (%)	Mean ± SD or n (%)	
Epidemiology				
Age (years)^a^	38.12 ± 12.58	39.84 ± 12.38	36.40 ± 12.79	0.339
Sex (female)^b^	41 (82.0%)	23 (92.0%)	18 (72.0%)	0.138
Saliva				
UFR (mL/min)^a^	1.06 ± 0.33	1.05 ± 0.25	1.06 ± 0.40	0.944
Salivary pH^a^	7.13 ± 0.41	7.06 ± 0.42	7.19 ± 0.40	0.271
Buffer capacity^a^	10.06 ± 0.87	10.24 ± 0.83	9.88 ± 0.88	0.144
Clinical factors				
Mask wearing time (hours)^a^	5.88 ± 2.93	5.60 ± 2.75	6.16 ± 3.13	0.505
Xerostomia^b^	14 (28.0%)	3 (12.0%)	11 (44.0%)	0.025*
Smoking^c^	3 (6.0%)	1 (4.0%)	2 (8.0%)	1.000
Candidiasis^c^	3 (6.0%)	2 (8.0%)	1 (4.0%)	1.000
Volatile sulfate compounds				
H_2_S (ppb)^a^	47.68 ± 105.32	42.64 ± 115.46	52.72 ± 96.23	0.739
CH_3_SH (ppb)^a^	10.84 ± 22.73	3.84 ± 10.57	17.84 ± 29.00	0.031*
VSC total (ppb)^a^	58.72 ± 123.14	46.48 ± 124.09	70.96 ± 123.48	0.488
CH_3_SH over 5 ppb^b^	15 (30%)	2 (8.0%)	13 (52.0%)	0.001**

a: Results were obtained using Mann–Whitney U test; b: chi-square test (two-sided); c: Fisher’s exact test (two-sided). *p* value was considered as significant when *p* value < 0.05 (*p < 0.05, **p < 0.01). The significant results are shown in bold.

### VSCs levels and cut-off values for halitosis

**Figure 1:**
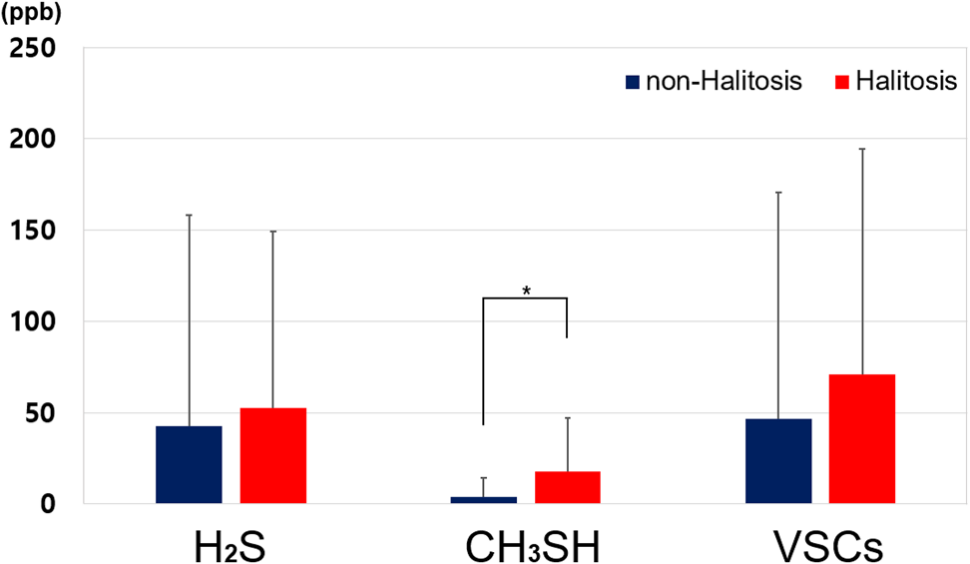
Comparison of H_2_S, CH_3_SH, and total VSCs between non-halitosis and halitosis groups.

**Figure 2:**
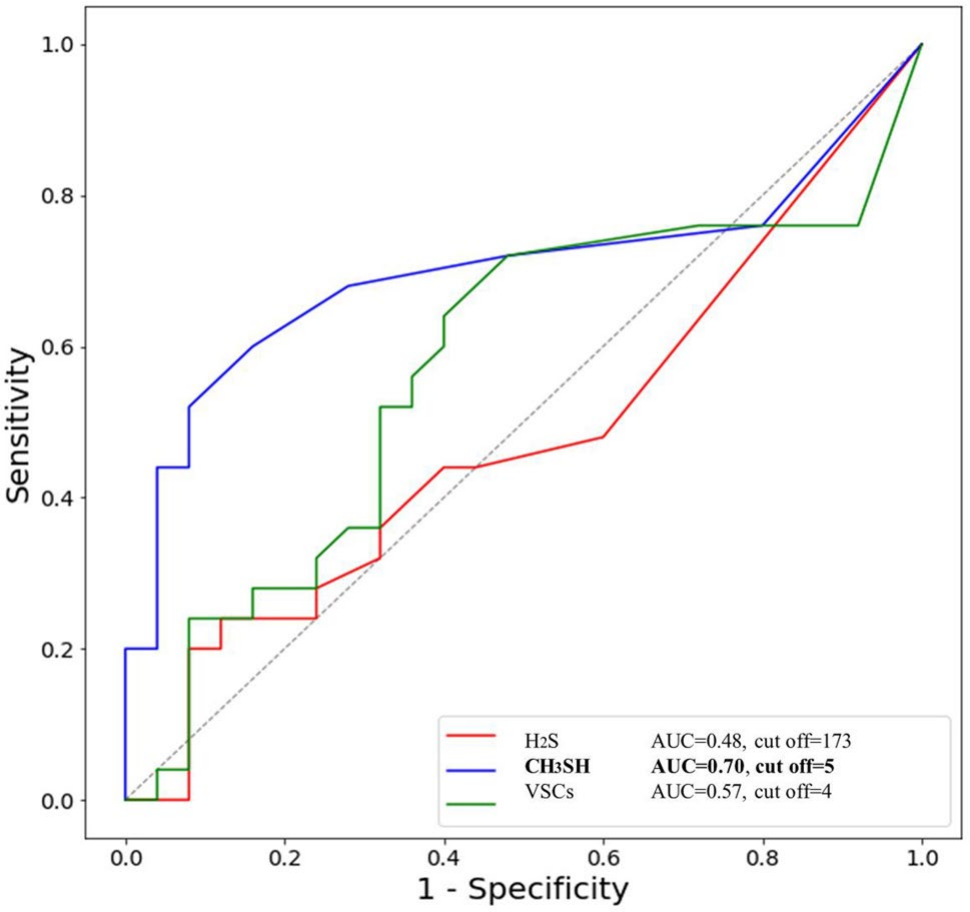
Cut-off values of VSCs for diagnosing self-perceived halitosis.

The mean value of CH_3_SH (methyl mercaptan) was significantly higher in the halitosis group than in the non-halitosis group (17.84 ± 29.00 vs. 3.84 ± 10.57 ppb, *p* = 0.031). However, the mean values of H_2_S and total VSC did not differ between the non-halitosis and halitosis groups (Fig. 1). In the total sample, Spearman’s correlation coefficient between CH_3_SH and H_2_S was 0.502 (*p* < 0.001), and it reached 0.734 in the halitosis group (*p* < 0.001). When the cut-off value of VSCs for diagnosing self-perceived halitosis was investigated based on the AUC, the CH_3_SH value was 0.70, which was an “acceptable discrimination” level, and its cut-off value was derived as 5 ppb (Fig. 2). Those who exceeded the cut-off value of CH_3_SH for self-perceived halitosis of 5 ppb were considered to have objective halitosis, and were observed in 52% of the halitosis group and 8% of the non-halitosis group, with a statistically significant difference (*p* < 0.01) (Table 1). However, predicting self-perceived halitosis by H_2_S level was not statistically significant.

### Total bacterial DNA amount and Shannon diversity index of saliva and mask

The total bacterial DNA copy numbers were significantly larger in the saliva sample than in the mask sample (2143714000.00 ± 2573528711.43 vs. 7758668.22 ± 29904469.06, *p* < 0.001), and the average value was 276 times greater in the saliva than in the mask. Among all 14 bacteria investigated, saliva was overwhelmingly more abundant than the mask sample (all *p* < 0.001). Shannon diversity index also significantly higher in saliva than in the inner surface of the mask (2.62 ± 0.81 vs. 1.15 ± 1.52, *p* < 0.001). The read numbers of bacterial DNA identified by RT-PCR of 14 bacteria are shown in Supplementary Table 1. The datasets generated and/or analyzed during the current study are available in the OSF repository [https://osf.io/mvwn6].

### Bacterial profile and halitosis

**Figure 3:**
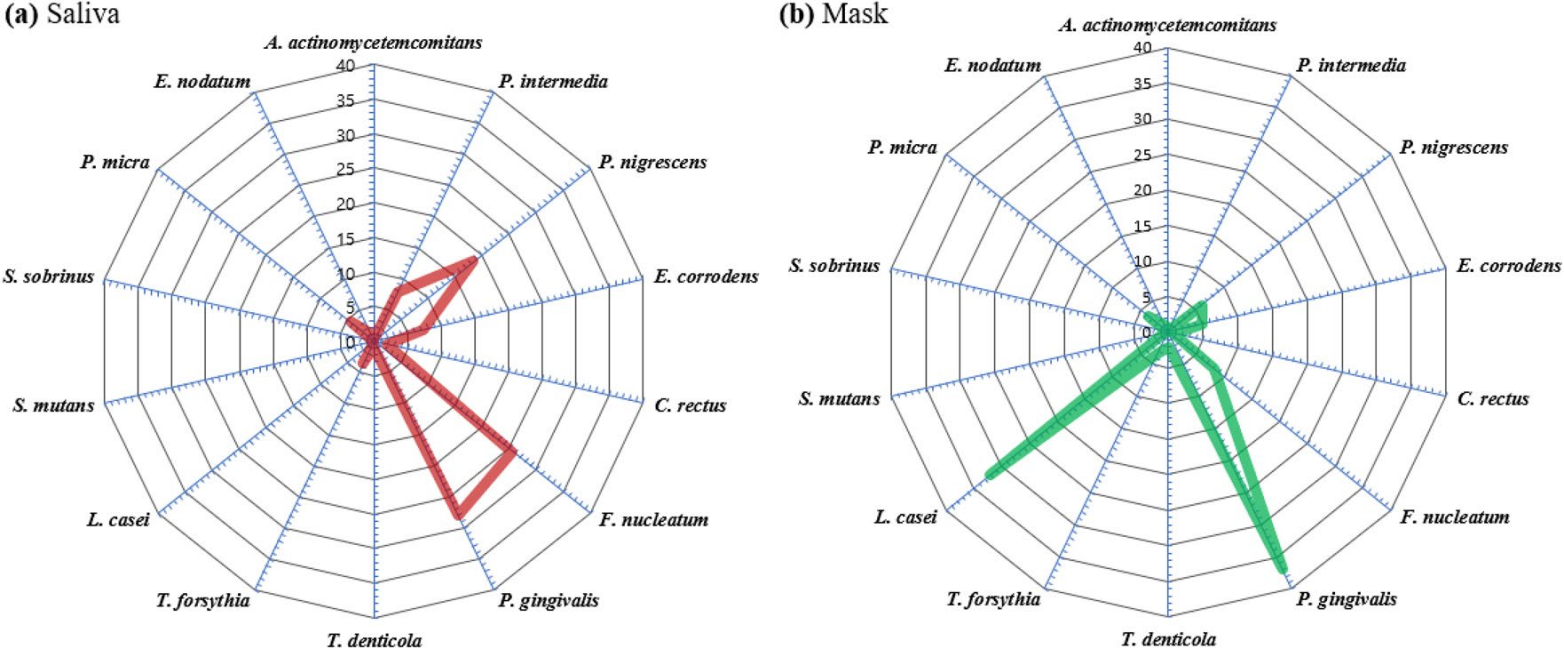
Bacterial profile in (**a**) saliva and (**b**) mask samples. Saliva: DNA copy number of each bacterium detected in saliva, mask; DNA copy number of each bacterium detected on the inner surface of the mask.

In saliva samples, the most frequently observed Gram-negative species, which had the highest DNA copy number, was *Pg*, followed by *Fn, Pn*, *Pi,* and *Ec*. Among the Gram-positive species in the saliva, *Pm* was the highest. In the mask sample, Gram-negative *Pg* was observed in the highest frequency. In the bacterial profiles of saliva and mask samples, different compositions were observed, except that *Pg* was the most frequently observed species (Fig. 3). When both the saliva and mask data were divided into halitosis and non-halitosis groups, interestingly, we found no significant differences between the DNA copy number of all 14 bacteria investigated, total bacterial DNA copy numbers, and Shannon diversity index (all *p* > 0.05). In other words, the bacterial profile did not differ significantly with the presence or absence of halitosis (Table 2).

**Table 2:** Composition and diversity of bacteria on the inner surface of the mask and unstimulated whole saliva.

	Saliva		*p* value of Saliva ^†^	Mask		*p* value of Mask ^‡^
	Non-Halitosis (n = 25)	Halitosis (n = 25)		Non-Halitosis (n = 25)	Halitosis (n = 25)	
Gram-negative species						
* Aa*	177281.68 ± 856921.09	9180.64 ± 33895.0168005072	0.337	0.00 ± 0.00	0.00 ± 0.00	NA
* Pi*	4821470.16 ± 13816176.03	2139538.60 ± 8123468.27	0.408	1802.76 ± 6731.83	417.20 ± 2086.00	0.334
* Pn*	6805631.68 ± 10080516.23	9339315.72 ± 17573555.26	0.535	3429.12 ± 7032.59	35862.69 ± 160126.38	0.322
* Ec*	1922641.91 ± 3096370.84	4408746.80 ± 9845312.84	0.238	6324.62 ± 19136.16	24620.91 ± 87469.88	0.316
* Cr*	371265.91 ± 884469.29	1397912.36 ± 3843152.19	0.204	172.40 ± 511.30	5037.83 ± 21824.26	0.276
* Pg*	5313643.46 ± 14357807.02	19282463.34 ± 51957255.87	0.206	8557.95 ± 26705.53	227628.89 ± 996170.35	0.283
* Td*	356665.05 ± 519337.09	480122.43 ± 1211563.71	0.643	1214.72 ± 4204.99	12587.52 ± 49802.06	0.266
* Tf*	846712.91 ± 1504287.25	2277485.64 ± 5385709.05	0.211	2312.97 ± 3801.42	17830.83 ± 69812.69	0.278
* Fn*	7010426.96 ± 8424698.62	15463094.83 ± 23593046.21	0.102	3123.43 ± 5845.64	49517.01 ± 209611.16	0.28
Gram-positive species						
* Lc*	806.58 ± 3353.26	30399.55 ± 141525.58	0.325	0.00 ± 0.00	205120.04 ± 1019891.99	0.325
* Sm*	246552.84 ± 1073173.54	55574.68 ± 119407.75	0.385	696.56 ± 1704.86	4218.78 ± 17740.75	0.333
* Ss*	2159.39 ± 10119.97	870.75 ± 3850.33	0.721	431.8 ± 2159.00	251.88 ± 1259.40	0.721
* Pm*	773465.99 ± 1844515.03	3096405.07 ± 8452786.41	0.191	1104.24 ± 3826.57	22412.05 ± 104552.19	0.319
* En*	775644.32 ± 2698126.85	300028.15 ± 925187.79	0.411	0.00 ± 0.00	6247.86 ± 29789.42	0.305
Total bacterial DNA	1716026311.29 ± 2253733724.43	2571947330.86 ± 2838493141.81	0.244	13687255.75 ± 41314936.65	1568893.83 ± 2107737.32	0.156
Shannon diversity index	2.58 ± 0.84	2.65 ± 0.79	0.758	0.95 ± 1.42	1.35 ± 1.61	0.364

Results were obtained via Mann–Whitney U test. *p* value was considered as significant when *p* value < 0.05.

†Comparison of the bacteria in saliva between non-halitosis and halitosis groups.

ǂComparison of the bacteria of the inner surface of the mask between non-halitosis and halitosis groups.

*Aa: Aggregatibacter actinomycetemcomitans; Pi, Prevotella intermedia; Pn, Prevotella nigrescens; Ec, Eikenella corrodens; Cr, Campylobacter rectus;*

*Pg, Porphyromonas gingivalis; Td, Treponema denticola; Tf, Tannerella forsythia; Lc, Lactobacillus casei; Fn, Fusobacterium nucleatum;*

*Sm, Streptococcus mutans; Ss, Streptococcus sobrinus; Pm, Parvimonas micra; En, Eubacterium nodatum.*

NA, not applicable.

### Correlation between bacteria in saliva and mask

**Figure 4:**
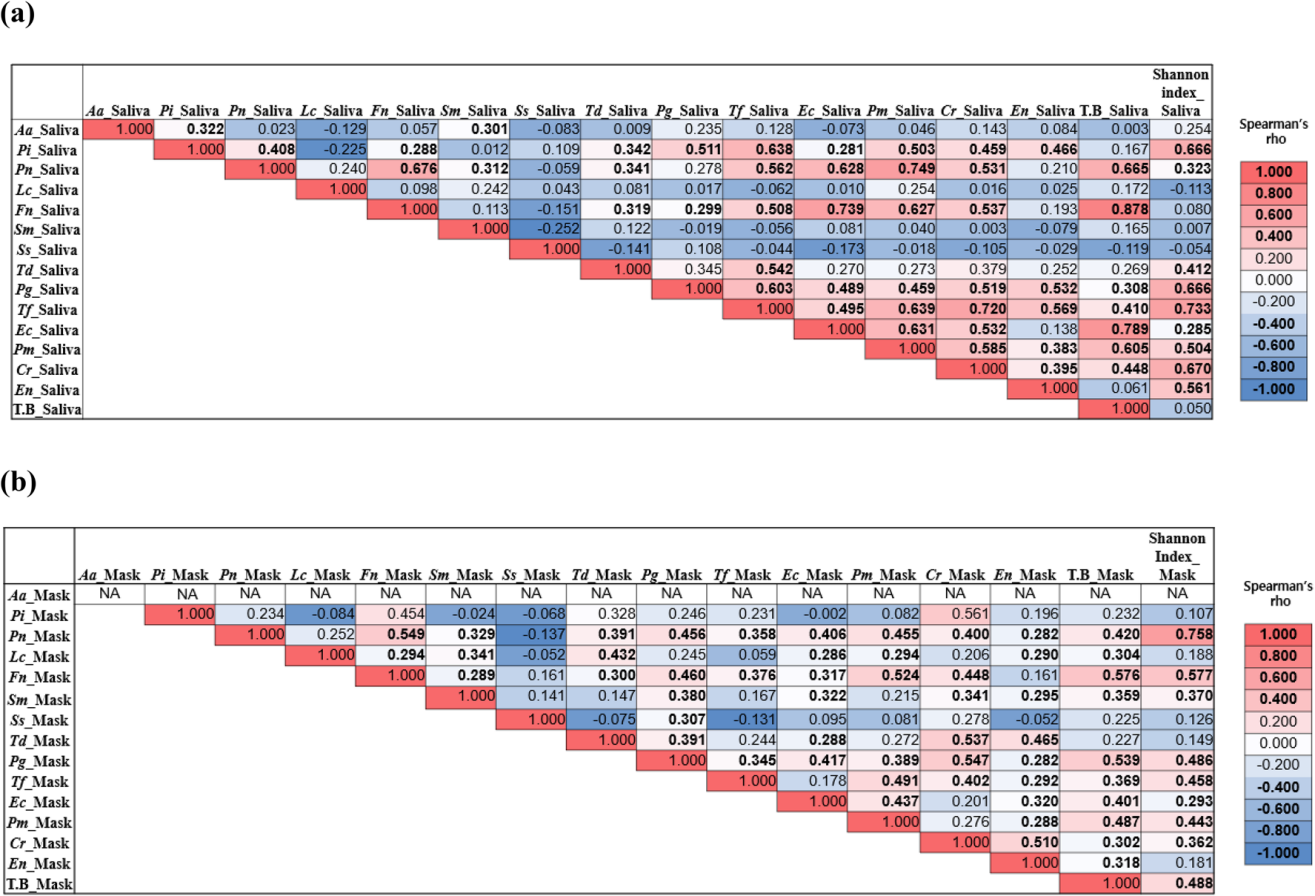
Bacterial correlation in (**a**) saliva and (**b**) mask samples. *Aa: Aggregatibacter actinomycetemcomitans; Pi: Prevotella intermedia; Pn: Prevotella nigrescens; Lc: Lactobacillus casei; Fn: Fusobacterium nucleatum; Sm: Streptococcus mutans; Ss: Streptococcus sobrinus; Td: Treponema denticola; Pg: Porphyromonas gingivalis; Tf: Tannerella forsythia; Ec: Eikenella corrodens; Pm: Parvimonas micra; Cr: Campylobacter rectus; En: Eubacterium nodatum;* T. B: total bacterial DNA copy numbers. NA: not applicable; *Aa* was not detected on the inner surface of the mask, and we could not examine the correlation between Aa_Mask and other bacteria.

Some bacteria were correlated with other bacteria in the saliva and mask samples (Fig. 4). The correlation coefficient (Spearman's rho) between the bacteria in saliva was larger than that of the mask sample, indicating a stronger correlation. In the saliva sample, bacteria with Spearman's rho ≥ 0.7 or higher were *Pn* and *Pm* (r = 0.749), *Fn* and *Ec* (r = 0.739), and *Tf* and *Cr* (r = 0.720) (all *p* < 0.001). On the other hand, a relationship with Spearman's rho ≥ 0.6 or higher was not observed between the bacteria in the mask sample. Furthermore, the bacteria-to-bacteria correlations varied from sample to sample. Significant positive correlations with Spearman's rho of 0.5 or higher were observed between *Pn* and *Fn* (r = 0.549), *Pg* and *Cr* (r = 0.547), and *Td* and *Cr* (r = 0.537) (*p* < 0.05). Significant positive correlations commonly observed in saliva and mask samples are *Pn–(Fn*, *Sm,* and *Td)*, *Pg–(Tf, Ec, Pm, Cr,* and *En)* relationships, *Fn–(Td, Pg, Tf, Ec, Pm,* and *Cr)* relationships, and *Pi–En* relationship. *Aa* did not exist in the mask sample; therefore, there was no correlation.

### Correlation between bacteria in the saliva and mask

**Figure 5:**
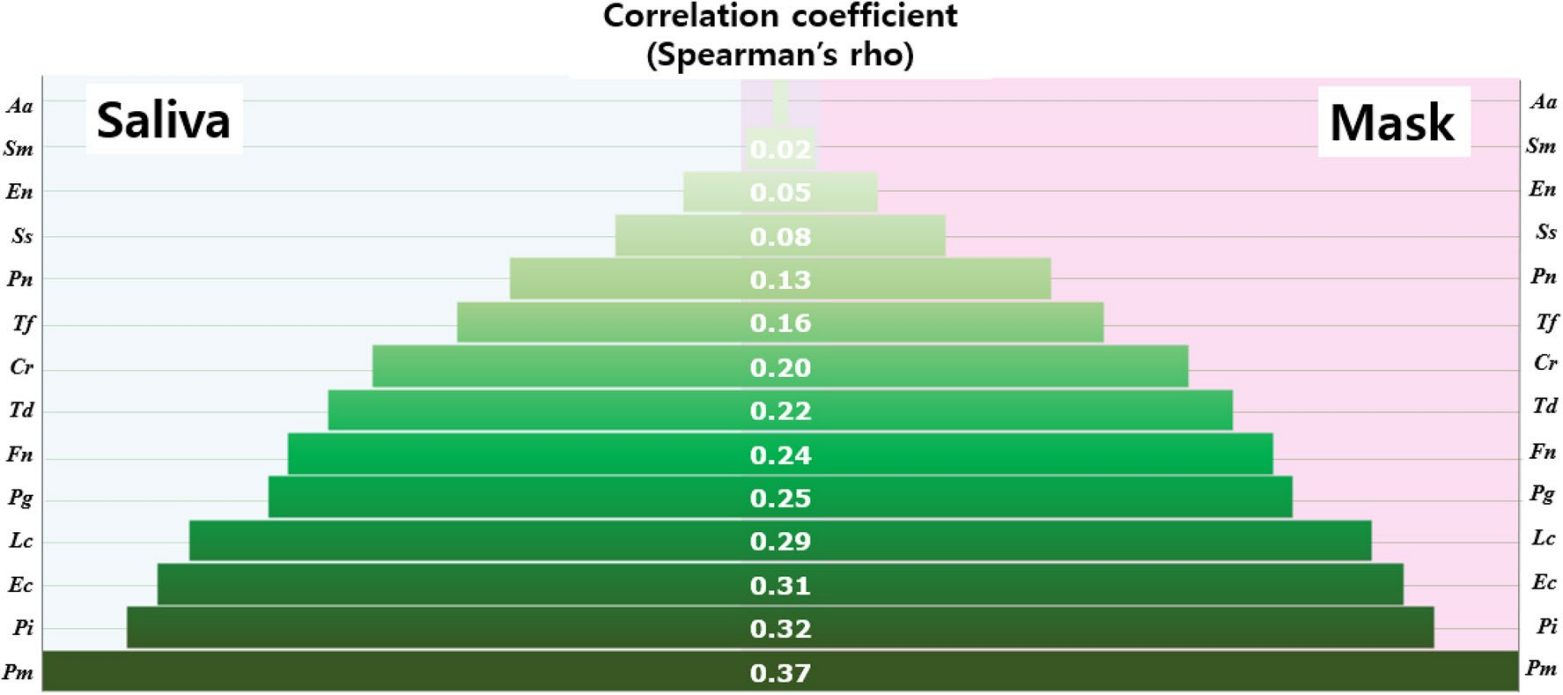
Arrangement in increasing order of correlation between bacteria in saliva and the inner surface of the mask. *Aa: Aggregatibacter actinomycetemcomitans; Sm, Streptococcus mutans; En, Eubacterium nodatum; Ss, Streptococcus sobrinus; Pn, Prevotella nigrescens; Tf, Tannerella forsythia; Cr, Campylobacter rectus; Td, Treponema denticola; Fn, Fusobacterium nucleatum; Pg, Porphyromonas gingivalis; Lc, Lactobacillus casei; Ec, Eikenella corrodens; Pi, Prevotella intermedia; Pm, Parvimonas micra.*

We investigated the origin of the bacteria found in the mask. The species with a significant positive correlation between saliva and mask samples were *Pi* (r = 0.324, *p* = 0.022) and *Ec* (r = 0.309, *p* = 0.029) among Gram-negative species, and *Pm* (r = 0.366, *p* = 0.009) and *Lc* (r = 0.293, *p* = 0.039) among Gram-positive species. That is, if these four species, *Pi, Ec, Pm,* and *Lc*, are abundant in saliva, there may also be many inside the mask (Fig. 5).

### Clinical factors related to the increase of VSCs

The most striking point when considering the correlation coefficient is that the VSC level increased as the mask-wearing time increased. In the halitosis group, mask-wearing time was strongly correlated with H_2_S (r = 0.896, *p* < 0.001), CH_3_SH (r = 0.939, *p* < 0.001), and total VSC (r = 0.854, *p* < 0.001) levels. Although the Spearman's rho level was low in the non-halitosis group, it was also strongly correlated with H_2_S (r = 0.801, *p* < 0.001), CH_3_SH (r = 0.831, *p* < 0.001), and total VSCs (r = 0.834, *p* < 0.001). Other clinical factors, including age, sex, smoking habits, xerostomia, and candidiasis, did not affect H_2_S, CH_3_SH, or total VSC levels. Salivary flow rate and buffer capacity were also not correlated with the VSCs levels. A decrease in salivary pH in the non-halitosis group was significantly correlated with an increase in CH_3_SH concentration (r = −0.451, *p* < 0.05) (Table 3).

**Table 3:** Correlations between volatile sulfur compounds and clinical factors.

		H_2_S	CH_3_SH	Age	Sex (Female)	Smoking	Mask wearing time	Salivary flow rate	Salivary pH	Salivary buffer capacity
Non-Halitosis group (n = 25)										
H_2_S	Spearman's rho	1.000	0.323	− 0.124	−0.360	0.264	0.801***	0.063	0.136	− 0.042
	*p *value		0.116	0.555	0.077	0.203	0.000	0.766	0.516	0.842
CH_3_SH	Spearman's rho	0.323	1.000	0.197	− 0.157	0.319	0.831***	− 0.137	0.451*	0.035
	*p *value	0.116		0.344	0.453	0.120	0.000	0.513	0.024	0.868
Total VSC	Spearman's rho	0.834***	0.699***	0.002	− 0.351	0.258	0.834***	0.065	0.346	− 0.026
	*p *value	0.000	0.000	0.994	0.085	0.214	0.000	0.759	0.090	0.902
Halitosis group (n = 25)										
H_2_S	Spearman's rho	1.000	0.734***	0.124	− 0.120	0.132	0.896***	0.103	0.084	− 0.262
	*p *value		0.000	0.554	0.568	0.528	0.000	0.625	0.690	0.206
CH_3_SH	Spearman's rho	0.734***	1.000	0.146	− 0.131	0.052	0.939***	0.007	− 0.117	− 0.193
	*p* value	0.000		0.487	0.533	0.807	0.000	0.973	0.578	0.356
Total VSC	Spearman's rho	0.854***	0.968***	0.122	− 0.131	0.051	0.854***	0.002	− 0.076	− 0.222
	*p *value	0.000	0.000	0.561	0.534	0.807	0.000	0.993	0.717	0.286

Results were obtained using Spearman’s correlation analysis. Differences were considered significant at *p* < 0.05 (*p < 0.05, **p < 0.01, ***p < 0.001). VSC: volatile sulfur compound level. Significant values are in bold.

### Bacterial factors related to increase of VSCs

**Figure 6:**
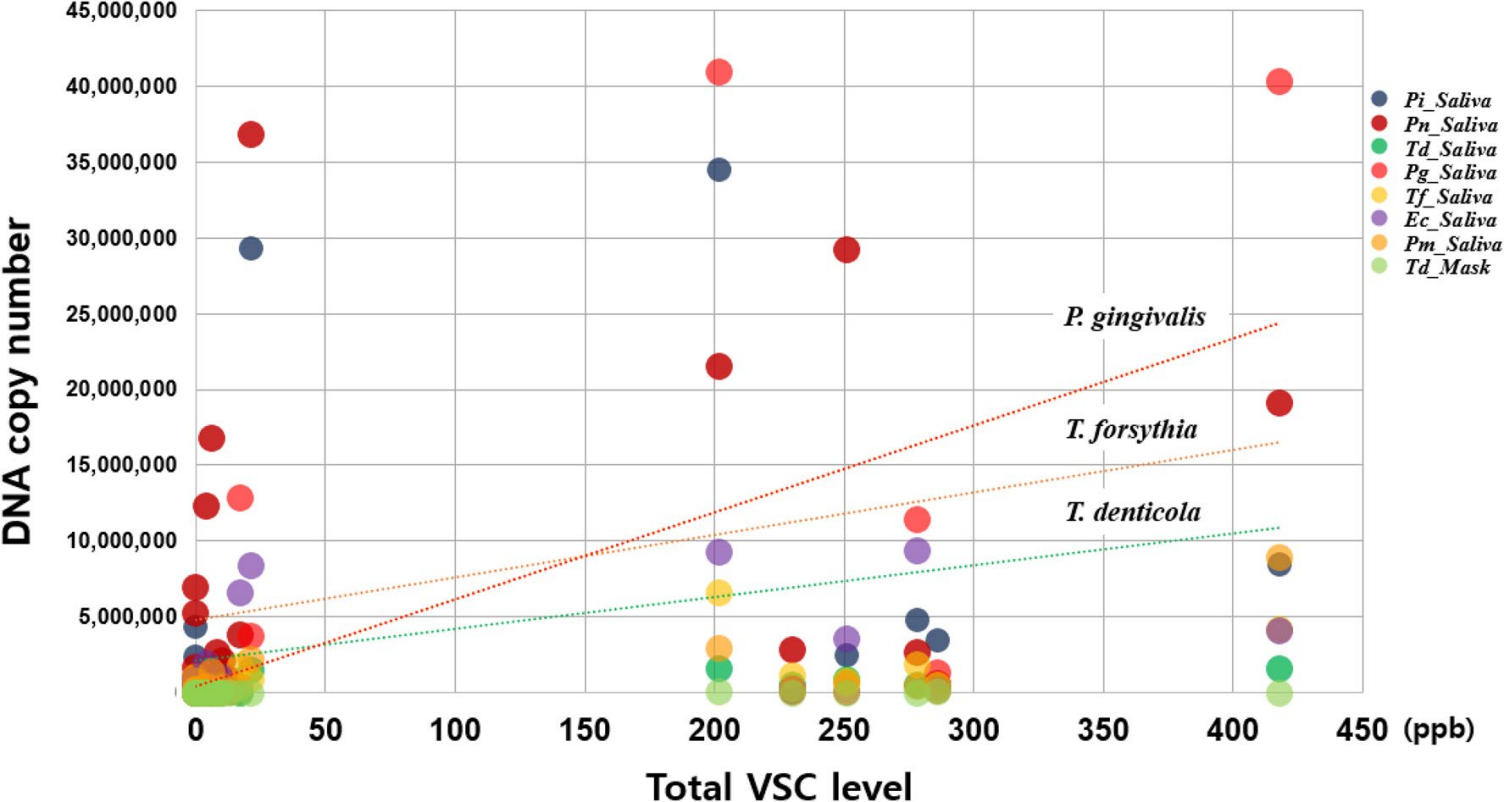
Bacteria positively correlated with increase in total VSC level. *Pi: Prevotella intermedia, Pn: Prevotella nigrescens, Td: Treponema denticola, Pg: Porphyromonas gingivalis, Tf: Tannerella forsythia, Ec: Eikenella corrodens, Pm: Parvimonas micra, _Saliva:* DNA copy number of each bacterium detected in Saliva*, _Mask:* DNA copy number of each bacterium detected on the inner surface of the mask.

Oral microorganisms in saliva had a greater effect on VSC levels than bacterial factors in the mask, and the amount of DNA in 9 out of 14 bacteria in the saliva sample, total bacterial DNA copy numbers, and Shannon diversity index of saliva were positively correlated with VSC level. In the halitosis group, H_2_S levels showed a significant positive correlation with the DNA copy number of *Tf* (r = 0.625, *p* = 0.001), *Pg* (r = 0.585, *p* = 0.002), *Pi* (r = 0.546, *p* = 0.005), *Pm* (r = 0.510, *p* = 0.009), *Ec* (r = 0.497, *p* = 0.012), *Pn* (r = 0.469, *p* = 0.018), *Fn* (r = 0.465, *p* = 0.019), *Td* (r = 0.465, *p* = 0.019), and *Aa* (r = 0.464, *p* = 0.020) in saliva in that order. CH_3_SH levels were significantly and positively correlated with *Pg* (0.594, *p* = 0.002), *Td* (r = 0.528, *p* = 0.007), *Tf* (0.492, *p* = 0.013), *Pi* (r = 0.474, *p* = 0.017), and *Pn* (r = 0.406, *p* = 0.044) in the saliva. In the halitosis group, H_2_S (r = 0.455, *p* = 0.022), CH_3_SH (r = 0.484, *p* = 0.014), and total VSCs (r = 0.509, *p* = 0.005) were positively correlated with the Shannon diversity index of the saliva. The total VSC level also showed a similar correlation pattern to CH_3_SH and had a positive correlation with *Pg* (r = 0.627, *p* < 0.001), *Tf* (r = 0.535, *p* = 0.003), *Td* (r = 0.517, *p* = 0.004), and *Pm* (r = 0.505. *p* = 0.005), *Pi* (r = 0.494, *p* = 0.006)*, Pn* (r = 0.414, *p* = 0.020), and *Ec* (r = 0.370, *p* = 0.034) (Fig. 6). Regarding the bacterial profiles of the masks, only *Td*_Mask was positively correlated with CH_3_SH (r = 0.414, *p* = 0.040) and total VSCs (r = 0.374, *p* = 0.033) in the halitosis group (Table 4).

**Table 4:** Bacterial factors that increase VSC levels.

Parameters		*Aa*_Saliva	*Pi*_Saliva	*Pn*_Saliva	*Fn*_Saliva	*Td*_Saliva	*Pg*_Saliva	*Tf*_Saliva	*Ec*_Saliva	*Pm*_Saliva	T.B_Saliva	Shannon diversity index_Saliva	*Td*_Mask
Non-Halitosis group (n = 25)													
H_2_S (ppb)	Spearman's rho	−0.073	0.459*	0.160	0.392	0.193	− 0.198	− 0.046	−0.015	0.154	0.266	− 0.369	− 0.200
	*p *value	0.729	0.021	0.445	0.053	0.355	0.343	0.826	0.943	0.462	0.199	0.069	0.337
CH_3_SH (ppb)	Spearman's rho	− 0.326	0.034	0.374	0.422*	0.011	− 0.058	0.179	0.298	0.249	0.408*	0.008	0.319
	*p *value	0.112	0.871	0.066	0.036	0.958	0.782	0.392	0.148	0.231	0.043	0.970	0.120
VSC total	Spearman's rho	− 0.242	− 0.293	0.288	0.545**	0.266	− 0.163	0.120	0.232	0.256	0.443*	− 0.229	0.070
	*p *value	0.122	0.078	0.082	0.002	0.099	0.218	0.284	0.132	0.109	0.013	0.136	0.370
Halitosis group (n = 25)													
H_2_S (ppb)	Spearman's rho	0.464*	0.546**	0.469*	0.465*	0.497*	0.585**	0.625**	0.497*	0.510**	0.490*	0.455*	0.354
	*p*-value	0.020	0.005	0.018	0.019	0.011	0.002	0.001	0.012	0.009	0.013	0.022	0.082
CH_3_SH (ppb)	Spearman's rho	0.209	0.474*	0.406*	0.301	0.528**	0.594**	0.492*	0.309	0.508**	0.404*	0.484*	0.414*
	*p *value	0.016	0.017	0.044	0.144	0.007	0.002	0.013	0.132	0.009	0.045	0.014	0.040
Total VSC	Spearman's rho	0.305	0.494**	0.414*	0.320	0.517**	0.627**	0.535**	0.370*	0.505**	0.420*	0.509**	0.374*
	*p* value	0.069	0.006	0.020	0.059	0.004	0.000	0.003	0.034	0.005	0.018	0.005	0.033

The results were obtained via Spearman’s correlation analysis. *P*-value was considered as significant when *p* value < 0.05 (*p < 0.05, **p < 0.01). The significant results are shown in bold. *Aa: Aggregatibacter actinomycetemcomitans, Pi: Prevotella intermedia, Pn: Prevotella nigrescens, Fn: Fusobacterium nucleatum, Td: Treponema denticola, Pg: Porphyromonas gingivalis, Tf: Tannerella forsythia, Ec: Eikenella corrodens, Pm: Parvimonas micra,* T.B: total bacterial DNA copy numbers, _Saliva: DNA copy number of each bacterium detected in Saliva, _Mask: DNA copy number of each bacterium detected on the inner surface of the mask, VSC: volatile sulfur compound level.

The increase in the VSC level was strongly correlated with *Pg*, *Td*, and *Tf* in the halitosis group, and *Pi* and *Fn* increased the VSC level in the non-halitosis group. In the non-halitosis group, H_2_S was positively correlated with *Pi_*Saliva (r = 0.459, *p* < 0.05), CH_3_SH was positively correlated with *Fn_*Saliva (r = 0.422, *p* < 0.05), total bacterial DNASaliva (r = 0.408, *p* < 0.05), total VSCs were positively correlated with *Fn_*Saliva (r = 0.545, *p* < 0.01), and total bacterial DNASaliva (r = 0.443, *p* < 0.05).

## Discussion

Halitosis is a term used to describe any undesirable odor in the exhaled air, whether the odorant is of intra-oral or non-oral origin. Halitosis is mainly caused by VSCs produced by the putrefactive action of oral microorganisms in the oral cavity, and a VSC level above the reference point is called genuine halitosis^2,16^. Among the important VSCs, the CH_3_SH levels were significantly higher in the halitosis group than in the non-halitosis group. When the cut-off value was set to 5 ppb CH_3_SH, the proportion of genuine halitosis in the halitosis group was 52 vs. 8%. That is, among individuals with self-perceived halitosis, 52% had genuine halitosis and 48% had pseudo-halitosis. In addition, 8% of individuals had genuine halitosis, even if they were unaware of halitosis. In a previous study that measured VSC levels using Halimeter (Interscan Corp., Chatsworth, CA, USA), the prevalence of halitosis was 78%, and self-awareness was reported only in 20.5%^16^. The main results of the present study support that long-term wearing of a mask may increase halitosis levels in individuals with self-perceived halitosis during the COVID-19 pandemic. The microorganisms existed not only in the oral cavity, but also inside the mask, and the bacterial species in some mask samples were derived from saliva. Salivary microorganisms, including *Pg*, *Tf*, *Td*, *Pm*, *Pi, Pn*, and *Ec*, are a major cause of oral malodor, and *Td* in masks can also cause bad breath.

Regarding salivary microorganisms, Gram-negative anaerobes represented by *Pg, Td*, and *Tf* particularly contributed to the increase in VSC levels. Halitosis has multiple etiologies. However, approximately 90% of the causes are intra-oral, and less than 10% have an extra-oral origin^20^. In the oral cavity, temperature and humidity could go up to 37 °C and 96% respectively, during oral exhalations. Thus, the oral cavity is a good habitat for microorganism growth, in which over 700 types of bacteria are found^21^. Among these, proteolytic obligate anaerobes and Gram-negative species are responsible for odorous compounds that can cause halitosis^16^. The development and progression of periodontitis are related to an imbalanced oral microbial community enriched with red-complex Gram-negative bacteria, including *Pg, Td*, and *Tf*^22^. Sigmund Socransky introduced the red complex concept in 1998, and periodontal diseases, along with poor oral hygiene and tongue coating, have been found to be common in people with halitosis^16,23,24^. In this study, people with a healthy periodontal status and moderate to good oral hygiene were targeted, but *Pg* and *Tf* in saliva and *Td* in both saliva and mask were the key causative agents for increasing VSC in people with self-perceived halitosis. Some healthy individuals with no history of periodontal diseases can develop halitosis because of the retention of bacteria in the oral cavity^25^. Further studies are required to clarify the oral malodor–periodontitis link and determine whether oral bacteria and their metabolism are key mediators in periodontitis patients. In healthy controls, *Pi, Fn*, and Gram-negative anaerobes were associated with an increase in VSC levels. *Pg* and *Pi* were prevalent in adult periodontitis^26^, and influenced the production of VSC^27^. *Fn* is a representative oral bacterium for halitosis^28^. Both Gram-negative and Gram-positive species were capable of generating VSCs, but the core species were Gram-negative.

The amounts of *Pi, Ec, Pm*, and *Lc* in the mask samples were correlated with those in the saliva samples, and *Pg* was the most frequently observed species in both samples, but the bacterial composition of the samples was different. The microorganisms inside the mask can originate from the oral cavity, upper respiratory tract, skin, and external environment. However, owing to the structure of the KF94 mask^29^, the proportion of bacteria from the external environment that penetrated the mask was low. It is difficult for bacteria (which are larger than viruses) to penetrate the mask, owing to pore size of masks that are small enough to inhibit the passage of viruses in any direction^30,31^. However, other nose or skin-derived microorganisms were not investigated in this study. Thus, our hypothesis that the microorganisms present on the inner surface of the mask were derived and proliferated from saliva and/or the oral cavity was partially supported by our results. The oral cavity has various microhabitats, including tongue biofilms, teeth, subgingival fluid, dental plaque, and saliva, and the microorganism profile and amounts differ depending on where it is sampled^24,32^. In a recent COVID-19 mask study, the number of bacterial colonies was greater on the face than on the outer-side^33^. This is probably because different bacteria have different shapes, metabolisms, surface adhesion, and survival strategies. Furthermore, the inside of the mask has elevated temperature and humidity, which are closely related to exhalation in a closed circuit^34^. This environment can induce the growth of certain anaerobic bacterial species. In this study, higher mask-wearing time was related to the increase in the VSC level. The selective proliferation of certain bacteria can lead to an increase in VSCs^35^. Although the effectiveness of masks against viral transmission has been extensively studied, few have reported on the potential health problems caused by bacteria adhering to the inner surface of masks.

Furthermore, the ability of bacteria to adhere to the inner surface of the mask depends on complex interactions between the bacteria themselves, other bacteria, and the host. *P. gingivalis* has high surface adhesion using fimbriae. Furthermore, *T. denticola* and *T. forsythia*, which consist of a red complex together, also present high adhesion ability^36–38^. *P. micra* attaches to oral epithelial cells and forms a synergistic biofilm with *F. nucleatum*^39^. *P. intermedia*, *E. corrodens*, and *L. casei*, which had a significant positive correlation between bacteria collected saliva and those from mask samples, and that also demonstrated surface adhesion in previous studies^40–42^. For delivery and migration from the oral cavity to the inner surface of the mask through the saliva droplets, the size and surface characteristics of the bacteria should be considered^43^. Unfortunately, few studies have systematically compared the differences in size and/or surface adhesion of these bacteria. In general, each bacterium possesses varying degrees of surface adhesion, and differences in dominant bacterial species ultimately lead to differences in the composition of biofilms or microbial communities^44^. Bacteria are social organisms that interact extensively within and between species in response to external environmental stimuli^45^. The view that halitosis is mainly caused by an imbalance in the composition of oral microorganisms, that is dysbiosis^46^, is gaining momentum. In our results, various correlations were found between Gram-negative and Gram-negative and even between Gram-negative and Gram-positive bacteria, and the types and strengths of these correlations were different in mask and saliva samples. Oral bacteria related to halitosis have been identified as *Actinomyces spp.*, *Bacteroides spp*., *Eubacterium spp*., *Fusobacterium spp*., *Porphyromonas spp., Prevotella spp., Selenomonas spp., Solobacterium spp., Tannerella forsythia,* and *Veillonella spp.,* which interact with each other^2,15^*. Pg* was the most abundant species in the mask and saliva samples, and several species, including *Pg* and *Td,* were associated with an increase in the VSC. Considering that *Pg, Td*, and *Tf* form the red complex, simultaneously creating a VSC, *Pg* is synergistic with either *Tf* or *Td* and induces host inflammation^47^. High VSC levels can cause lethal toxicities and act as pathogens and carcinogens in the host cells^48^. These results suggest that when using a mask in the COVID-19 pandemic situation, in the event of oral malodor, therapeutics and management strategies considering bacterial interactions, hygiene of the inner surface of the mask, and replacement of the mask are necessary.

This study had several limitations. First, the number of samples was small compared with that of the 14 bacterial species investigated. Numerous multicenter studies are required to confirm these results. Secondly, the statistical analysis method was centered on the comparison between the mean, proportion, and correlation. A more sophisticated statistical analysis will be required in the next step, considering that bacteria and factors related to the increase in VSC interact with each other. Lastly, because the breakthrough innovation of the oral microorganism analysis method was not accomplished, the clinical trial will be verified later based on state-of-the-art next-generation sequencing techniques. However, to the best of our knowledge, this study is unique because it proved that the long-term use of masks during the COVID-19 pandemic was associated with halitosis, which can be related to an increase in certain Gram-negative microbes. The results were derived by comprehensively investigating oral microorganisms and clinical factors. Wearing a well-fitting mask and practicing social distancing, along with immunizations, have helped to reduce the spread of the SARS-CoV-2 virus and protect people worldwide. The results of the present study support the long-term, continuous use of masks with the potential to cause bodily harm in addition to halitosis caused by oral bacteria. The trajectory of COVID-19 and repetition of the pandemic are difficult to predict^49^. Therefore, governments and societies globally should be able to recommend wearing masks based on our results.

## Conclusion

Here, we investigated the VSC levels of subjects with halitosis and the related-oral microbial profile of the inner surface of KF94 masks. We identified which oral microbiome compositions increase VSC levels and which bacteria are associated with this change in saliva and masks. CH_3_SH levels were significantly higher in the halitosis group than in the non-halitosis group, but there was no significant group difference in the H_2_S value. In the halitosis group, total VSC levels had a positive correlation with the bacterial count, in the order of *Pg*, *Td*, *Tf*, *Pi*, and *Pn*. *Td* was strongly correlated with CH_3_SH and total VSCs in the mask’s bacterial profile, but only in the halitosis group. Mask wearing time showed a strong correlation with total VSCs, H_2_S, and CH_3_SH levels. Thus, the mask-wearing time was a major factor associated with VSC level increases. During the COVID-19 pandemic, the VSC levels of subjects wearing masks were associated with increased amounts of oral bacteria, particularly Gram-negative anaerobes such as *Pg* and *Td*. Our results suggest that individuals with halitosis can control these Gram-negative bacteria by improving oral hygiene with mechanical tooth brushing to remove biofilms or by antibacterial gargling and changing their masks regularly.

## Materials and methods

### Study population

A total of 50 healthy subjects participated voluntarily (45 women; mean age, 38.12 ± 12.58 years) at the Kyung Hee University Dental Hospital between August 1, 2021, and October 31, 2021, and the group consisted of 25 subjects with self-perceived halitosis (mean age, 39.84 ± 12.38 years) and 25 subjects with non-halitosis (mean age, 36.40 ± 12.79 years). For the sample size calculation, G*Power software (latest ver. 3.1.9.7; Heinrich-Heine-Universität Düsseldorf, Düsseldorf, Germany) was used and a total of 25 subjects were calculated, and a total of 50 subjects (25 subjects for each group) were recruited. The research protocol for this study was reviewed in compliance with the Declaration of Helsinki and approved by the Institutional Review Board of Kyung Hee University Dental Hospital in Seoul, South Korea (KHD IRB, IRB No-KH-DT21023). Informed consent was obtained from all participants. All subjects were asked to complete questionnaires used to analyze sex, age, presence of self-perceived halitosis, xerostomia, candidiasis, smoking habit, and mask wearing time. We recruited subjects until each group comprised 25 subjects. Salivary flow rate, pH, and buffer capacity were investigated using a series of protocols. A dental mirror and periodontal probe (Probe UNC 15; Hu-Friedy, Chicago, Il, USA) were used by the examiner (LYH) to evaluate the teeth, periodontal tissue, and oral mucosa. Systemic health and disease status were investigated using elaborate questionnaires. The inclusion criteria were as follows: medically healthy adults over the age of 20 with a healthy periodontal condition, who continuously wore the KF94 mask for more than 3 h, having lost < 2 teeth in the permanent dentition, able to voluntarily read and judge the consent form, and able to participate in the study. The following cases were excluded: (1) have taken medications that may affect salivation (antibiotics, anti-inflammatory drugs, anticonvulsants, immunosuppressants, calcium channel blockers, psychiatric medications, etc. within the last 6 months; (2) have re-used the mask wore the day before; (3) have uncontrolled systemic disease; (4) are pregnant or breastfeeding; and (5) are wearing intraoral appliances or complete removable dentures or partial dentures. In the case of insufficient data collection, individuals who dropped out by themselves or due to the research situation were also excluded.

### Saliva collection and salivary flow rate, pH, and buffer capacity

Unstimulated whole saliva samples (saliva samples) were collected for 10 min using the spitting method between 15:00 and 17:00 to minimize diurnal variability. The unstimulated salivary flow rate (UFR) was expressed in mL/min. All participants refrained from consuming alcohol the previous day and were instructed to abstain from eating, drinking, and brushing their teeth before the saliva sample collection. To determine salivary pH and buffer capacity, GC Saliva Check Buffer kits (GC, Tokyo, Japan) were used. After measuring UFR, a salivary pH test strip was placed in the resting unstimulated whole saliva sample for 10 s. A pH value of ≥ 6.8, corresponds to healthy saliva, whereas a value < 6.6 is characterized as acidic. To measure saliva buffering capacity, three areas of the test strip were dampened with saliva using a pipette. After 2 min, the colors were observed, and scores of 4, 3, 2, 1, and 0 were attributed to green, green/blue, blue, red/blue, and red, respectively. The result was interpreted using the scheme in the kit, where each resulting total value corresponds to a degree from “very low” to “normal” salivary buffer capacity (minimum 0 points, maximum 12 points), as follows:0–5: very low; 6–9: low; 10–12: normal.

### VSC measurement

For participants in the validation sample, H_2_S and CH_3_SH levels in mouth air were measured using a portable gas chromatographer (TwinBreasor II, IsenLab, Gyeonggido, Korea) equipped with a flame photometric detector. As a 10 mL sample of participant’s mouth air passed through an electrolytic sensor, the concentrations of H_2_S and CH_3_SH were detected, indicating a peak level in ppb on the digital scale of the monitor. The concentrations of H_2_S and CH_3_SH and their sum (total VSCs) are expressed in parts per billion (ppb).

### Bacterial DNA extraction and 16S ribosomal RNA gene sequencing

Fourteen anaerobic species were investigated. Nine Gram-negative anaerobes [*Aggregatibacter actinomycetemcomitans (Aa)*, *Prevotella intermedia (Pi)*, *Prevotella nigrescens (Pn)*, *Eikenella corrodens (Ec)*, *Campylobacter rectus (Cr)*, *Fusobacterium nucleatum (Fn), Porphyromonas gingivalis (Pg)*, *Treponema denticola (Td)*, and *Tannerella forsythia (Tf)*] and five Gram-positive anaerobes [*Lactobacillus casei (Lc)*, *Streptococcus mutans (Sm)*, *Streptococcus sobrinus (Ss)*, *Parvimonas micra (Pm)*, and *Eubacterium nodatum (En)*] were included. The strength of scientific evidence varies, but 14 species have been found to be related to halitosis. The total bacterial DNA, bacterial profile, and individual taxonomic abundance of oral bacterial species were determined. 16S rDNA primers were used to amplify the V1-V3 region. Mask samples of the bacteria were collected by washing with saline the inside of the KF94 mask worn by the subjects for more than 3 h. The KF94 mask blocked approximately 94% of the fine particles, including viruses and bacteria, in the air.

### Bacterial DNA isolation

Saliva and mask samples were vortexed vigorously, and 500 μL of each sample was added to corresponding tubes containing 500 μL of lysis buffer (5 mM EDTA, 5 M guanidine hydrochloride, and 0.3 M sodium acetate). After vortexing to mix the sample with the lysis buffer, the tubes were incubated at 65 °C for 10 min. S2 buffer (0.25 g/mL silicon dioxide; Merck KGaA, Darmstadt, Germany) was thoroughly mixed by vortexing, and 20 μL of this buffer was added to the sample–lysis buffer mixture. After vortexing, the tubes were incubated for 5 min at room temperatures between 40 and 140 °F with intermittent inversion. The mixture was then centrifuged at 5,000 rpm for 30 s, and the supernatant was carefully removed. One milliliter of PureLink (Invitrogen Corporation, Carlsbad, CA, USA) PCR purification washing buffer 1 (50 mM 3-[N-morpholino] propane sulfonic acid buffer, pH 7.0, with 1 M sodium chloride) was activated by adding 160 mL of 100% ethanol and then added to the tubes and vortexed until the beads were resuspended completely. After centrifugation at 5000 rpm for 30 s, the supernatant was carefully removed, and 1000 μL of washing buffer 2 (ethanol) was added and vortexed to completely resuspend the beads. Finally, the tubes were centrifuged at 5000 rpm for 30 s, and the supernatant was removed completely. One hundred microliters of elution buffer (100 mM Tris–HCl, pH 7.5; 1 M EDTA) was added to the tube and vortexed to resuspend the beads. The tubes were then incubated at 65 °C for 10 min to dissolve the DNA and separate it from the beads. After centrifugation at 13,000 rpm for 5 min, the supernatant was transferred to a sterile microcentrifuge tube and subjected to PCR.

### Real-time PCR amplification

Real-time PCR amplification was performed for each sample using primers specific for the aforementioned 14 species of oral bacteria. Bacterial 16S ribosomal RNA (rRNA) primers were used to quantify total bacteria. The reaction mixture consisted of 10 μL of 2X Master-mix (GeNet Bio, Daejeon, Korea), 2.5 pM forward and reverse primers, and 5 μL DNA template. The total reaction volume (20 μL) was subjected to qPCR amplification under the following conditions: pre-denaturation at 95 °C for 10 min, followed by 45 cycles of denaturation at 95 °C for 15 s, and annealing/extension at 60 °C for 1 min. The synthesized plasmid DNA from each bacterium served as a positive control, whereas DNase/RNase-free water was used as a negative control.

### α-Diversity according to the Shannon diversity index

α-Diversity was calculated using the Shannon diversity index and bacterial richness was measured as the total number of bacterial DNA copies. The α-diversity levels of the microbial profiles were compared using Shannon index calculations using the following formula:

$$ {\text{H}} = {-}\sum {\text{pi}} \times {\text{ln}}\left( {{\text{pi}}} \right) $$

where pi is the relative abundance, H is the Shannon diversity index, pi is the proportion of cells of the ith species in the whole community, and pi = n/N, where n is the number of cells of a given taxon/species, and N is the total number of bacterial cells in a community. The minimum value of the Shannon diversity index was zero, indicating no diversity; thus, the greater the value, the higher the diversity. In real-world ecological data, the Shannon diversity index typically ranges from 1.5 to 3.5, and rarely reaches 4.5^50^.

### Statistical analysis

In the present study, various statistical methods were used for data analysis. First, the absolute and percentage distributions of all nominal and categorical variables, and the means and standard deviations, were obtained, and descriptive data analysis was performed. The bacterial profile, total bacterial DNA amount, and Shannon diversity index of oral microorganisms in the saliva and mask samples were investigated. The results for the halitosis and non-halitosis groups were compared. The Mann–Whitney U test was used to investigate differences in the mean values related to the oral microbiome between the two groups. To analyze the distribution of discontinuous data, we used the χ^2^ test, Fisher’s exact test, and Bonferroni test for equality of proportions. Spearman’s correlation analysis was used to determine correlations between variables. Spearman’s correlation coefficient was expressed as Spearman’s rho (r), and when it was closer to the absolute value of 1, it indicated a stronger correlation. To show the performance of the classification model at the classification threshold, a receiver operating characteristic curve (ROC) curve was plotted, and the area under the ROC curve (AUC) value was calculated for each model. As the rule of thumb for interpreting the AUC value, AUC = 0.5 (no discrimination), 0.6 ≥ AUC > 0.5 (poor discrimination), 0.7 ≥ AUC > 0.6 (acceptable discrimination), 0.8 ≥ AUC > 0.7 (excellent discrimination), and AUC > 0.9 (outstanding discrimination)^51^. Statistical significance was set at *p* < 0.05. The data were analyzed using IBM SPSS Statistics for Windows (version 26.0; IBM Corp., Armonk, NY, USA).

### Informed consent

Informed consent was obtained from all the subjects involved in the study.

### Institutional review board statement

The research protocol for this study was reviewed in compliance with the Declaration of Helsinki and was approved by the Institutional Review Board of Kyung Hee University Dental Hospital in Seoul, South Korea (KHD IRB, IRB No-KH-DT21023). Informed consent was obtained from all participants.

## Supplementary Information

Supplementary Information 1.

Supplementary Information 2.

## Data Availability

The datasets generated and/or analyzed during the current study are available in in the OSF repository [https://osf.io/mvwn6]. The datasets used and/or analyzed during the current study are available from the corresponding author upon reasonable request.
